# The 75–99 C-Terminal Peptide of URG7 Protein Promotes α-Synuclein Disaggregation

**DOI:** 10.3390/ijms25021135

**Published:** 2024-01-17

**Authors:** Jany Dandurand, Magnus Monné, Valérie Samouillan, Martina Rosa, Alessandro Laurita, Alessandro Pistone, Donatella Bisaccia, Ilenia Matera, Faustino Bisaccia, Angela Ostuni

**Affiliations:** 1 CIRIMAT Physique des Polymères, Université Toulouse 3, Paul Sabatier, 118 Route de Narbonne, 31062 Toulouse, France; jany.lods@univ-tlse3.fr (J.D.); valerie.samouillan@univ-tlse3.fr (V.S.); 2 Department of Sciences, University of Basilicata, 85100 Potenza, Italy; magnus.monne@unibas.it (M.M.); martinarosa1999@gmail.com (M.R.); alessandro.laurita@unibas.it (A.L.); alessandro.pistone@unibas.it (A.P.); ilenia.matera@unibas.it (I.M.); 3 IRCCS Istituto Tumori “Giovanni Paolo II” of Bari, 70124 Bari, Italy; donibisaccia@gmail.com

**Keywords:** URG7, peptides, α-synuclein, bioinformatics, clathrate-like structures, intermolecular β-sheets

## Abstract

Up Regulation Gene seven (URG7) is the pseudogene 2 of the transporter ABCC6. The translated URG7 protein is localized with its single transmembrane α-helix in the endoplasmic reticulum (ER) membrane, orienting the N- and C-terminal regions in the lumen and cytoplasm, respectively, and it plays a crucial role in the folding of ER proteins. Previously, the C-terminal region of URG7 (PU, residues 75–99) has been shown to modify the aggregation state of α-synuclein in the lysate of HepG2 cells. PU analogs were synthesized, and their anti-aggregation potential was tested in vitro on α-synuclein obtained using recombinant DNA technology. Circular dichroism (CD), differential scanning calorimetry (DSC), Fourier-transform infrared (FTIR) spectroscopy, and microscopic techniques were used to assess the sample’s behavior. The results show that the peptides studied by themselves are prone to clathrate-like structure formation of variable stability. Aggregation of α-synuclein is accompanied by desolvation of its peptide chain and an increase in intermolecular β-sheets. The PU analogs all interact with α-synuclein aggregates and those possessing the most stable clathrate-like structures have the highest disaggregating effect. These findings suggest that the C-terminal region of URG7 may have a role in interacting and modulating α-synuclein structures and could be used to generate interesting therapeutic candidates as disaggregators of α-synuclein.

## 1. Introduction

Previous studies carried out in our and other laboratories have allowed the characterization of the structure and the endoplasmic reticulum (ER) localization of URG7, which is a protein comprising 99 amino acids initially identified to be over-expressed upon hepatitis B virus X antigen induction [1,2]. This protein was revealed to be expressed from the pseudogene 2 of the gene corresponding to the ABCC6 transporter of 1503 amino acids, in which mutations cause Pseudoxanthoma Elasticum [3,4]. The first 75 amino acids of URG7 and ABCC6 are identical, and this segment contains one transmembrane helix. URG7 is localized in the ER membrane with the N-terminal region in the lumen and the C-terminal region in the cytoplasm [1]. Experiments carried out on HepG2 cells treated with tunicamycin have shown that cells over-expressing URG7 are more resistant to the stress induced by tunicamycin by presenting a different unfolded protein response (UPR) with increased survival indicators compared with pro-apoptotic ones. Even the quantity of misfolded proteins decreases, suggesting a role for URG7 in protein folding typical of ER proteins [2]. Recently, differential calorimetry experiments have demonstrated that the C-terminal region of URG7 is able to disaggregate α-synuclein in HepG2 cell lysate [5].

α-synuclein is present in neurons in different conformational and oligomeric states, but the soluble oligomers and protofibrils, which are formed early in the fibrillation process, are particularly toxic for dopaminergic neurons, in which membrane damage, mitochondrial defects, and synaptic dysfunction are some examples of the proposed pathogenic mechanisms in Parkinson’s disease and other synucleinopathies [6,7,8,9]. Heat shock proteins (HSPs) and proteins from endoplasmic reticulum play an important role in maintaining cellular protein homeostasis and can protect neurons from damage by degradation of aggregates through different mechanisms [10,11]. Therefore, in the search for new therapeutic strategies, α-synuclein aggregation and misfolding are considered a priority target for drug development [12,13,14]. 

Peptides are well suited to be evaluated as potential aggregation inhibitors and/or potential α-synuclein disaggregators, thus reversing the ongoing neurodegeneration [15]. Most of the peptides proposed are fragments/or mutated fragments of the NAC (non-amyloid-β- component) domain as well as of the N-terminus of the α-synuclein or pre-NAC domain [16,17,18], the NAC-region and C-terminal region from a peptide library spanning the entire α-synuclein sequence [19], a larger library [20,21] and purified from natural sources, such as the peptide YIAEDAER from the marine snail *Neptunea arthritica cumingii* [22]. Established by segmenting all proteins deposited in the Protein Data Bank, the database proposed in [23] is centered on sequences adopting β-structures, specifically those identical or very similar to the regions of α-synuclein that are involved in aggregation. In addition, a more rational identification of peptides based on structure–activity relationship showed that also α-helical peptides of different origins, which do not interact with the functional monomeric α-synuclein, bind toxic oligomers and fibrils [24].

In this study, we have investigated how the C-terminal peptide of URG7 and its analogs may interact and disaggregate α-synuclein. Upon comparing the sequence of the C-terminal cytoplasmic region of URG7 with sequences of known anti-aggregating peptides, a potential β-strand forming motif was found that may interfere with α-synuclein oligomerization. Peptides containing this URG7 motif and variants of it were synthesized and tested in α-synuclein aggregation assays, showing how this rational approach may yield potent anti-aggregation peptides. These findings have the potential to be used in the design of new therapeutic strategies employing URG7-derived peptides to prevent or reverse the aggregation of proteins implicated in synucleinopathies.

## 2. Results

### 2.1. Design of Peptides Interfering with α-Synuclein Aggregation Based on URG7

**Figure 1:**
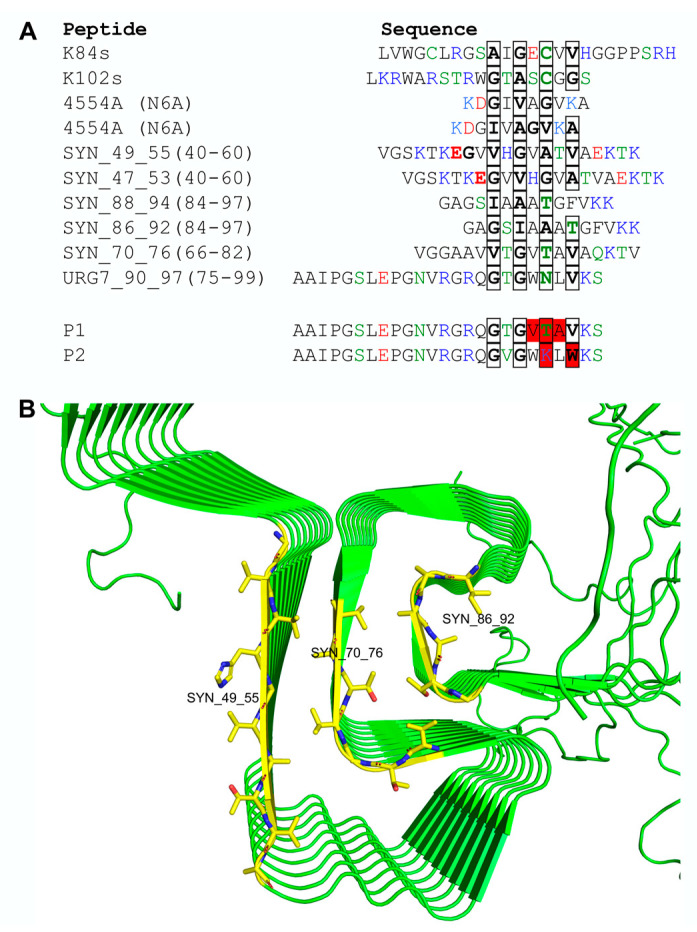
Design of peptides interfering with α-synuclein aggregation. (**A**) Sequence alignment of anti-aggregation peptides, α-synuclein β-strand-forming polymerization peptides, C-terminal peptide of URG7 (PU) and variants. The residue numbering is given for the small side chain pattern region (boxed parts) with the sequence displayed in parenthesis. Mutations made in the P1 and P2 peptides with respect to PU are indicated in red. (**B**) Structure of α-synuclein polymer filament formed by interactions of stacks of the Greek key motif of β-strands of single protomers [25]. The peptides forming the β-strands of the motif are shown in sticks with carbons in yellow.

Since the C-terminal peptide of URG7 (called PU, residues 75–99) has been shown to modify the aggregation state of α-synuclein [5], we decided to analyze this peptide and its possible relationship to α-synuclein in more detail. PU was compared to the anti-aggregation peptides 4554A(N6A), K84s, and K102s [17,20] and internal peptides of α-synuclein forming the aggregation state Greek key motif [25]. None of these sequences share any significant sequence identity or similarity; however, they all contain patterns of residues with short side chains that may be aligned so that alternating residues have a short side chain (Figure 1A).

Notably, the α-synuclein peptide 70–76 has short side chains also in the intervening positions of this pattern. This peptide is situated at the center of the Greek key motif when α-synuclein polymerizes into amylogenic filaments (Figure 1B). It forms a β-strand with its side chains making contacts on both sides with the side chains of two other β-strands (49–55) and (86–92) in the vicinity, forming the core of the Greek key motif. Unlike the central β-strand 70-76 of α-synuclein, the two flanking strands (49–55 and 86–92) and the anti-aggregation peptides clearly have the pattern with short side chains on one side of their β -strands but more polar, charged, or large side chains on the other side. 

The residues with short side chains in PU are most similar to those of α-synuclein 70–76 on one side of the peptide, whereas on the other side, it has larger W and L residues. This observation led us to imagine that if PU would take the place of the central Greek key peptide 70–76 of α-synuclein, it would be accommodated well against a flanking β-strand on one side, most probably against α-synuclein 86–92 but would interfere with the β-strand interaction on the other side. One or several such interactions would, therefore, obstruct the polymerization of α-synuclein since the Greek key β-strands of each protomer are stacked upon those of the other protomers, forming long parallel β-sheets (Figure 1B).

Based on this hypothetic mechanism for anti-aggregation peptide action, we mutated PU to make it more similar to α-synuclein peptide 70–76 and called P1 (Figure 1A) with the purpose of increasing its potential of substituting peptide 70–76 in the aggregates. PU was also mutated to make it incompatible with β-strand interaction, as in the α-synuclein Greek key motif, by disrupting the alternating small side chain pattern and introducing large side chains called P2 (Figure 1A), rendering the peptide less likely to bind to α-synuclein but if it does it would interfere even more with α-synuclein polymerization. As a positive control, the effective anti-aggregation peptide 4554A(N6A) (Figure 1A) [17], which is called P3 in this work, was chosen.

### 2.2. Assays for Structural Characterization of α-Synuclein Aggregation

**Figure 2:**
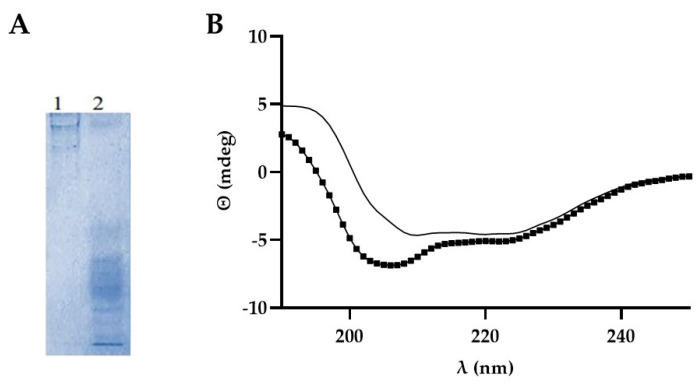
(**A**) Not-denaturing polyacrylamide gel. α-synuclein just solubilized (1) or heated at 45 °C (2) was analyzed by not-denaturing polyacrylamide gel and stained with Coomassie Blue; (**B**) CD spectra of α-synuclein S (—) and HS45 (▪) were recorded on a Jasco J-185 CD spectrophotometer (Easton, MD, USA) using a 0.1-cm cylindrical quartz cell.

In order to verify the potential disaggregating activity of the designed peptides, recombinant α-synuclein, prepared and purified by affinity chromatography according to [5] and showed in Supplementary Figure S1, was subjected to heating and stirring. Native gel electrophoresis confirmed that heating and stirring led to different states of aggregation of α-synuclein (Figure 2A). Analysis of the secondary structures of the protein just solubilized (S) and continuously stirred for 2 h at 45 °C (HS45) was carried out by circular dichroism (Figure 2B). Analysis of spectra for the evaluation of secondary structure content was performed with DichroWeb using the CONTINLL algorithm [26]. HS45 protein showed higher β-structures (9 and 29% of strand and turns, respectively) content than the unheated counterpart (11 and 20% of strand and turns, respectively). Compositions of 42% and 44% were the α-helix content of HS45 and S, respectively.

For further calorimetric and vibrational experiments, α-synuclein was solubilized in PB to a concentration of 700 µM. A fraction of this solution was centrifuged at 13,000 rpm/min for 10 min at 20 °C, resulting in an initial state of α-synuclein solutions. Another fraction of the solution was heated to 45 °C for different time intervals before centrifugation, resulting in heated state α-synuclein solutions; in all cases, experiments were performed on supernatants.

**Figure 3:**
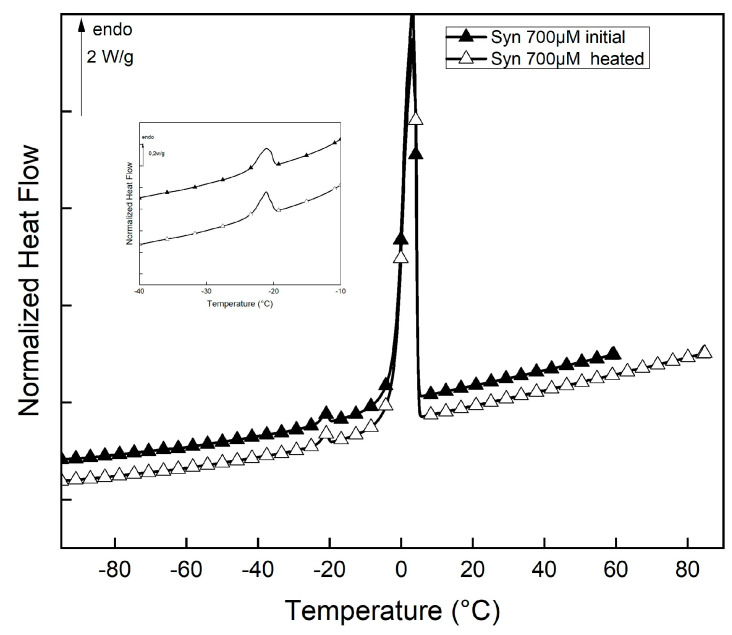
DSC thermograms of α-synuclein solutions (initial and heated at 45 °C for 2 h). The endothermic peak of the eutectic melting in the enlarged [−40; −10 °C] zone.

DSC (differential scanning calorimetry) measurements were carried out to investigate the thermal signature and the physical structure of α-synuclein solutions in the initial state (just solubilized) and after heating and stirring at 45 °C for 2 h. The thermograms obtained for both solutions are shown in Figure 3.

The DSC heating curve of α-synuclein solutions is characterized by two reversible peaks. As already reported on highly hydrated biological systems [5,27,28], the endothermic peak close to −20 °C is assigned to eutectic melting, while the intense endothermic peak close to 0 °C corresponds to the melting of primary ice [29]. The characteristics of these two transitions for both kinds of solutions are presented in Table 1.

An increase in the enthalpy of fusion of primary ice is observed for heated solutions of α-synuclein. As the amount of bulk water is proportional to this melting enthalpy of fusion [30], we can conclude that buried water or confined water is converted into bulk water during heating. These results demonstrate that the aggregation of α-synuclein is accompanied by desolvation of the peptide chains.

**Figure 4:**
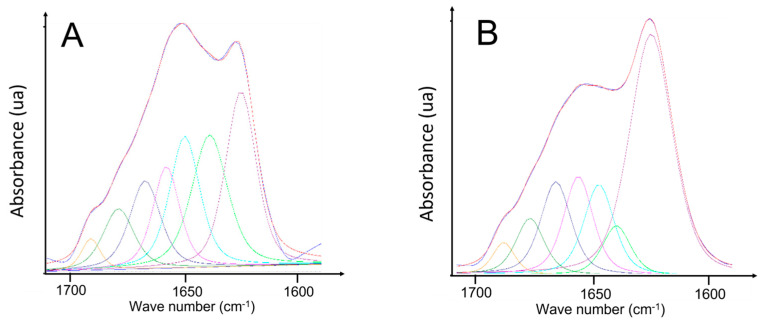
FTIR spectra in the [1600–1700 cm−^1^] and curve fitting of the amide I signal of (**A**) unheated α-synuclein (**B**) heated α-synuclein at 45 °C for 2 h.

FTIR spectroscopy was used to assess the quantity of the different secondary structures of α-synuclein in the initial state and the heated state after incubation at 45 °C for different time intervals via the decomposition of the amide I signal (see Section 4.7). FTIR spectra in the [1600–1700 cm^−1^] zone and curve fitting of the Amide I of unheated and heated α-synuclein are shown in Figure 4. The amide I spectrum of unheated α-synuclein (Figure 4A) is characterized by a main broad band close to 1650 cm^−1^ associated with random coil/water [31,32,33,34] and a shoulder at 1623 cm^−1^ characteristic of β-sheet structures. The shape of the amide I spectrum is consistent with a monomeric structure for α-synuclein.

After heating (Figure 4B), the amide I signal is changed drastically; the 1620 cm^−1^ band indicating the presence of intermolecular β-sheet is increased strongly (from 23.8% to 45.3%). This rise in intermolecular β-sheet structures is the hallmark of an aggregation process.

**Figure 5:**
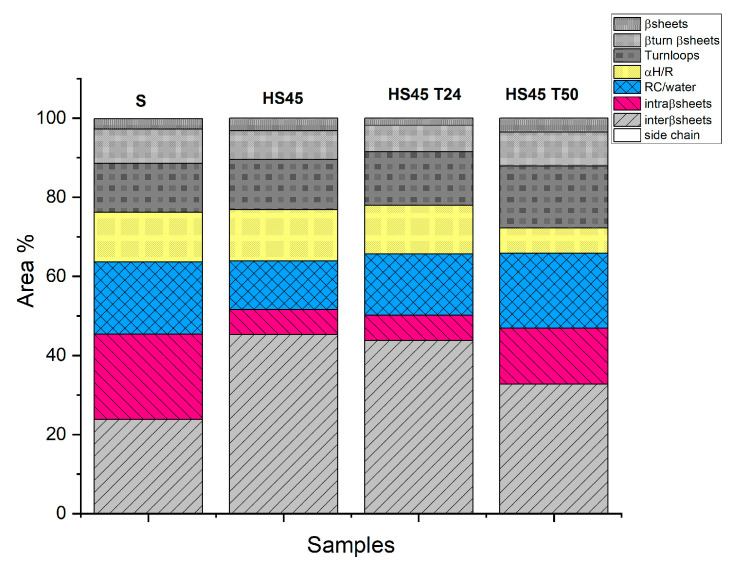
Quantification of the different secondary structures according to their different wave-number absorption for unheated α-synuclein and α-synuclein heated at 45 °C for different time intervals (HS45: 2H; HS45 T24: 24H; HS45 T50: 50H) (from amide I decomposition).

Molecular configuration changes were monitored by evaluating the proportion of different secondary structures of α-synuclein as a function of temperature and time (Figure 5). 

β-sheets structures absorb both high and low wave numbers, but only the low wave number components can be used to discriminate between inter- and intra-molecular β sheets. The amount of intermolecular β-sheets structures strongly increased in the first hours of incubation, and intramolecular β-sheets and random coil/water bands decreased. The proportions of different structures were maintained constant at 24 h and probably induced by aggregation forms, such as oligomers or fibrils. After 50 h of incubation, the proportion of intermolecular β-sheets and α-helix /water structures decreased as intramolecular β-sheets and random coil water increased, evidencing aggregated polymorphic fibrils.

The first increase in intermolecular β-sheets tracked by FTIR is driven by contact formation and water exclusion, also evidenced by the conversion of confined water into bulk water (DSC experiments). 

### 2.3. Circular Dichroism Analysis and Hydration of the Peptides

**Figure 6:**
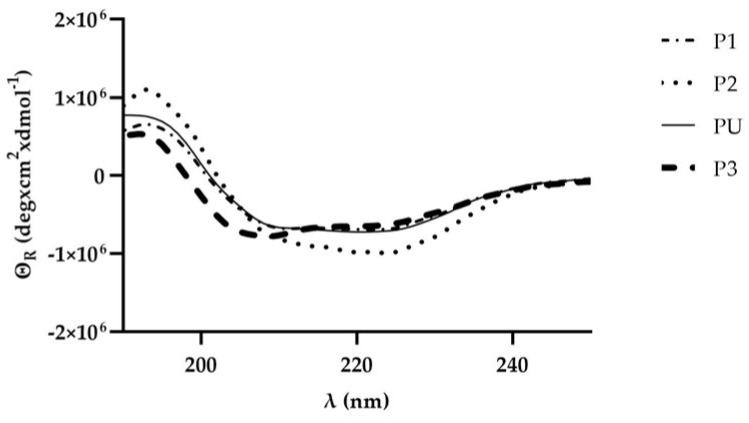
CD spectra of peptides were recorded on a Jasco J-185 CD spectrophotometer using a 0.1-cm cylindrical quartz cell on 7 µM peptides in PB.

Structural characterization of secondary structures of peptides was performed by circular dichroism (Figure 6). Analysis of spectra for the evaluation of secondary structure content was performed with DichroWeb using the CONTINLL algorithm. PU, P1 e P2 showed both around 50% of α-helix and β-strand. Although, due to the low number of amino acid residues, it was not possible to perform secondary structure analysis using DichroWeb, the P3 profile was very similar to others.

**Figure 7:**
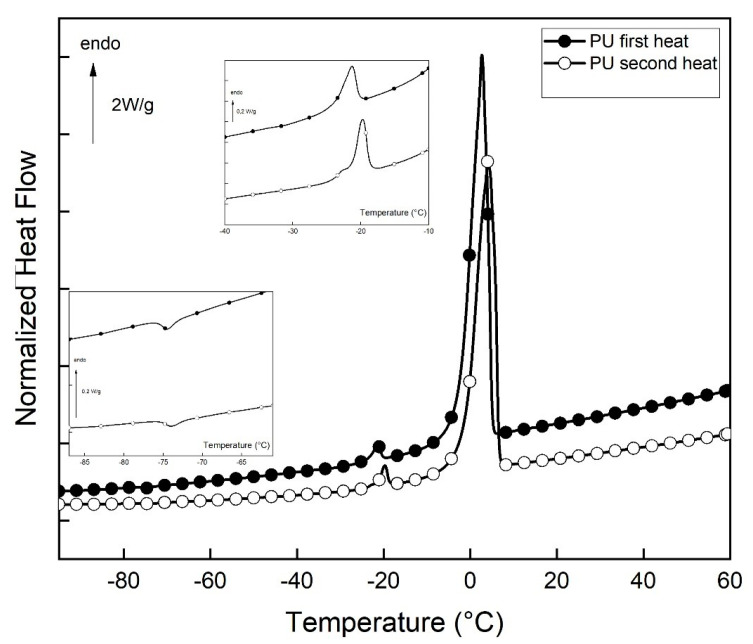
DSC thermograms of PU solution (first and second heat) were recorded. Exothermic transition in the enlarged [−100; −40 °C] zone. The endothermic peak of the eutectic melting in the enlarged [−40; −10 °C] zone.

Peptides (P1, P2, P3, and PU) were solubilized in PB to a concentration of 700 µM, and solutions were centrifugated at 13,000 rpm/min for 10 min at 20 °C. Further experiments were performed on supernatants. The thermograms obtained from the PU solution are shown in Figure 7.

DSC heating curves of PU are characterized by three reversible peaks. As reported [28], the low-temperature exothermic peak corresponds to the formation of a clathrate-like structure and is closely associated with the hydrophobic hydration of the peptide. Similar to other highly hydrated biological systems [5,27,28], the endothermic peak around −20 °C is assigned to eutectic melting, while the intense endothermic peak corresponds to the melting of primary ice [29]. P1, P2, and P3 solutions present similar features (DSC thermograms not shown). The thermal characteristics of the three transitions for all studied peptide solutions are presented in Table 2.

All the peptides studied are hydrophobically hydrated, as evidenced by the formation of clathrate-like structures. This hydrophobic structure is created at a higher temperature for the P1 solution than for the other ones, suggesting a difference in stability. It must be noted that P3, which is not an analog of PU and is known for its disaggregating effect [17], also possesses hydrophobic hydration.

### 2.4. Interaction between Peptides and Aggregated α-Synuclein

**Figure 8:**
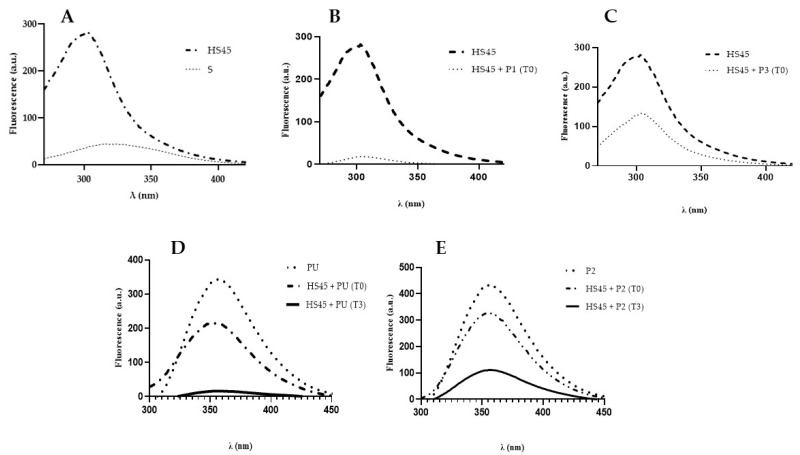
Fluorescence emission spectra of synuclein and peptides. (**A**) Fluorescence spectra at 264 nm excitation of α-synuclein (70 μM) just dissolved in PB (S) and kept with stirring and heating at 45 °C (HS45); (**B**) Fluorescence spectra at 264 nm of HS45 and immediately after addition of P1 (T0), after 3 h no fluorescence is recorded; (**C**) Fluorescence spectra at 264 nm of HS45 and immediately after addition of P3 (T0), after 3 h no fluorescence is recorded. (**D**) Fluorescence spectra at 275 nm of PU alone and after addition of HS45 immediately (T0) and after 3 h (T3); (**E**) Fluorescence emission spectra at 275 nm excitation of P2 alone and upon addition of α-synuclein (T0) and after 3 h (T3). The peptides were used at a concentration of 105 μM.

Tryptophan is the most fluorescent amino acid in proteins, followed by tyrosine for tryptophan-free proteins. Phenylalanine has weak fluorescence and is seldom used in protein studies [35]. α-Synuclein, lacking tryptophan residues, shows no fluorescence when excited at 275 nm; however, upon excitation at 264 nm, it shows a fluorescence spectrum with an emission maximum at 320 nm, presumably attributed to the presence of tyrosine and phenylalanine residues. In particular, it was observed that, at 264 nm excitation wavelength, the intrinsic α-synuclein fluorescence increased and shifted towards blue (from 320 nm to 305 nm), suggesting a higher exposure of the aromatic residues (phenylalanine and tyrosine residues) in a more hydrophobic environment (Figure 8A). The intrinsic fluorescence of HS45 was quenched upon the addition of the peptides P1 and P3 (which do not contain aromatic amino acids) (Figure 8B,C). The intrinsic fluorescence of the PU and P2 peptides, due to the presence of two tryptophan residues excited at 275 nm, exhibited an emission maximum of around 360 nm; therefore, changes in their fluorescence spectrum may be indicative of the interaction with α-synuclein itself (Figure 8D,E). All considered, the results suggested that peptides showed an ability to affect the three-dimensional structure of α-synuclein and then interact with it.

**Figure 9:**
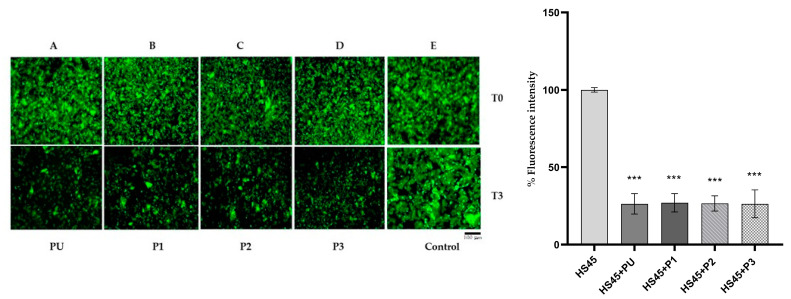
Representative fluorescence micrographs and quantitative analysis of fluorescence intensity of α-synuclein. Thioflavin (50 μM) was added to α-synuclein HS45 (25 μM). The images were acquired with a fluorescence microscope using the green light filter (510–560 nm) immediately after the addition of thioflavin (from **A**–**E**, T0) and at 3 h (T3) by the addition of PU, P1, P2 and P3, respectively. Control indicates a sample in which, instead of peptide, PB was added immediately (T0) and at 3h. Scale bar in black, below image is 100 μm. Magnification 20×. Data indicated for quantitative analysis were obtained normalized to HS45(T3) set to 100% and are presented as the mean ± SEM of four independent experiments. Statistical analysis was performed using Student’s *t*-test; *** *p* < 0.001.

The potential disaggregation effect of peptides was investigated using the Thioflavin-T (ThT) aggregation assay. No fluorescence of thioflavin was observed when it was added to freshly solubilized α-synuclein. On the contrary, fluorescent aggregates were observed when thioflavin was added to heated α-synuclein (Figure 9A–D). Upon addition of the peptides (T0), the fluorescence of the aggregates did not change but, over time (up to 3 h, T3), decreased by approximately 25% in all α-synuclein samples in the presence of each of the peptides. As a control, no change in fluorescence was observed up to 3 h if, instead of peptide, PB was added (Figure 9E). These results suggest that peptides are potential disaggregators of α-synuclein.

**Figure 10:**
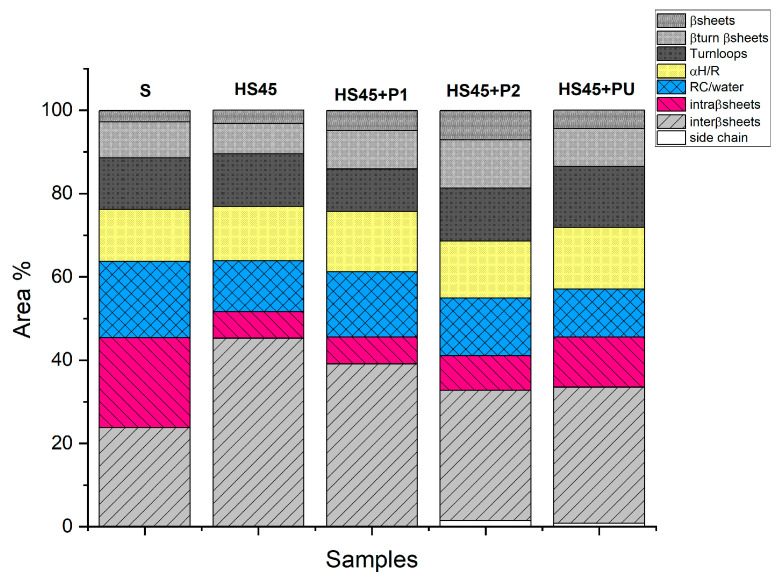
Quantification of the different secondary structures according to their different wave-number absorption for unheated/heated α-synuclein and (heated α-synuclein)/peptide to evaluate the disaggregating effect of peptides (From amide I decomposition).

The quantification of the secondary structures of unheated/heated α-synuclein and their evolution with the addition of peptides by FTIR analysis is shown in Figure 10. 

**Figure 11:**
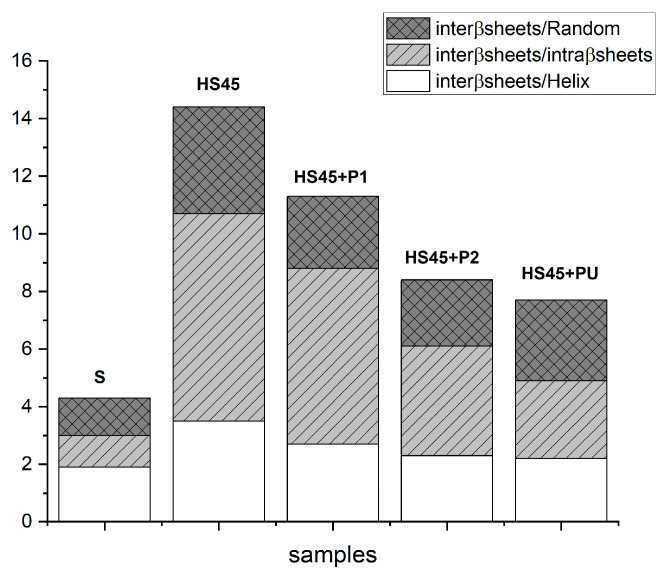
Quantification of the disaggregation effect of peptides (from Amide I decomposition).

When peptides were added to previously heated α-synuclein HS45 solutions, the amount of intermolecular β-sheets decreased in all cases and are associated with an increase in intramolecular β-sheets for P2 or PU addition. The amounts of random coils and α-helices were almost unchanged. The intermolecular β sheets/intramolecular β-sheets ratio (Figure 11) was drastically reduced for heated α-synuclein subsequently treated with P2 or PU, demonstrating their disaggregating effect.

**Figure 12:**
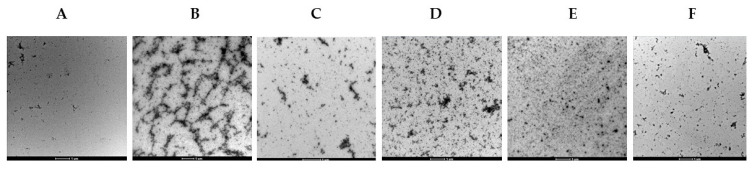
TEM micrographs of α-synuclein structures. (**A**) just solubilized; (**B**) heated and stirred α-synuclein, HS45; (**C**) HS45 after addition of PU; (**D**) HS45 after addition of P2; (**E**) HS45 after addition of P1; (**F**) HS45 after addition of P3. All images except B (captured at 9500×) were captured at 11,500× magnification.

TEM analysis showed very few and small aggregates in just solubilized α-synuclein (A) compared to heated α-synuclein sample (B); in addition, aggregates decreased when peptides were added (Figure 12). These results confirmed the potential disaggregation ability of the peptides.

## 3. Discussion

We have taken a rational approach to investigate the possible mechanism by which the C-terminal region of URG-7 interferes with α-synuclein aggregation, as previously observed in HepG2 cell lysate [5]. The effects of peptide variants of this region were assessed in an in vitro assay with recombinant α-synuclein incubated in conditions leading to a highly aggregated state (Section 2.2). These conditions were similar to what has been described previously in various experimental systems in which α-synuclein exhibits different aggregation states, fibrils, and oligomers [36,37,38]. The thermal analysis of the peptides and α-synuclein gave information on protein–water interactions and interfacial water and enabled all changes to be measured over a wide range of temperatures similar to previously reported methods [27,39]. The protein conformation and water interactions of wild-type and mutant α-synuclein have been shown to play a key role in its folding and aggregation processes [40,41,42,43,44]. FTIR spectroscopy allowed us to evaluate the contents of intermolecular β-sheets, which are correlated with the ultrastructural states of α-synuclein since parallel β-sheets are associated with fibrillar states and antiparallel β-sheets are associated with oligomeric states [31,45,46,47]. Red or blue shift processes, as well as a decrease in fluorescence intensity, are indicative of conformational changes in a protein/peptide containing fluorophores and interactions with molecular quencher, respectively [48]. Changes in the structure of α-synuclein upon heating or due to interaction with peptides are evident from intrinsic fluorescence analysis and the thioflavin assay. Moreover, also TEM showed qualitative evaluation of the α-synuclein aggregation states in various conditions. Thus, it was possible to follow what was happening to the interacting molecules by taking this multiple-technique approach to assess the conformational properties of their initial isolated, mixed changing, and resulting states.

The rationale of the design of the analogs of PU (residues 75–99 of the URG7 C-terminal region) was based on the hypothesis that an internal segment with alternating small side-chain residues in the C-terminal region inserted between the different molecules of α-synuclein during aggregation and thus distorting fibril growth or preventing it from happening (Section 2.1). Furthermore, this segment of PU presents a tryptophan, which could repel the interaction with the other β-strand located in the center of the Greek key motif and thereby further destabilize α-synuclein aggregation formation. PU was modified to be incorporated better into the center of the Greek key motif by substitution of the tryptophan and leucine with short side-chain residues, which could distort fibril growth without a repellant effect (P1). P2, on the other hand, was taken into consideration as a negative control because it might not bind to α-synuclein by having larger side chains on both sides of the presumed β-strand, which creates a greater steric hindrance for Greek key motif formation, but if it does bind to α-synuclein, it would effectively prevent aggregation through an increased repellant effect. P3, which is a 10-residue mutated peptide originating from α-synuclein, was chosen as a positive control because it has been shown to prevent the aggregation of α-synuclein and have disaggregating capacity [17]. 

The peptides P1, P2, P3, and PU isolated in aqueous solution were shown to contain mostly α-helical structures with very little content of β-strand by CD and clathrate-like formations by thermal analysis (Section 2.3). In biomolecules, the clathrate-like water is governed by three factors: hydrophobicity of the environment, nanoconfinement, and suitable anchoring groups. This structure disappears when one of these factors is lost, and two factors are needed to maintain it, namely the hydrophobic environment and confinement [49]. These results suggest that in solution, part of the peptides form α-helices and expose hydrophobic residues that order surrounding water molecules and might be prone to interaction with other hydrophobic entities. 

When P1 was mixed with the α-synuclein aggregates, the clathrate-like structure was lost (Supplementary Table S1), but intermolecular β-sheet content was almost constant (Figure 10 and Figure 11); however, disaggregation effects were observed on aggregated α-synuclein by the fluorescence emission spectra and micrographs (Figure 8, Figure 9 and Figure 11). We interpret these observations as P1 masking its hydrophobic residues by being inserted into the α-synuclein aggregates, which partly prevents the formation of larger aggregates or longer filaments.

In PU- and P2-treated aggregated α-synuclein, the clathrate-like structures of the peptides were conserved (Supplementary Table S1), whereas the content of intermolecular β-sheets was notably decreased (Figure 10 and Figure 11). We could note that the clathrate-like structures appeared at higher temperatures compared to those in solutions with only the peptides, which shows that the hydrophobic hydration state is changed. These results demonstrate that P2 and PU modify α-synuclein-water interactions and have a disaggregating effect, which is a result corroborated by the fluorescence spectra, micrograph, and TEM observations (Figure 8, Figure 9 and Figure 11). Based on these results, we deduce that PU and P2 interact with α-synuclein aggregates and strongly promote their disaggregation, but their exposed hydrophobic residues are not incorporated in continuous α-synuclein aggregates but remain accessible, maybe at the terminal ends of the filaments. As seen by these conclusions, the clathrate-like structures seem an interesting factor for monitoring the effects of the peptides on α-synuclein disaggregation. 

The results shown here are basically in line with the initial hypothesis, on which the PU peptides were designed, and the mechanism by which the C-terminal region of URG-7 interferes with α-synuclein aggregation, although they do not confirm them in detail because alternative explanations cannot be excluded. The specific variants P1 and P2 all regard changes in the residues between 94 and 97 of PU, and their different behaviors with α-synuclein demonstrate that this region plays a role in α-synuclein aggregate disaggregation. Supposedly, the protomers of the intermolecular β-sheet filaments of aggregated α-synuclein are in a constant association–disassociation equilibrium shifted towards association, and the peptides change this equilibrium. P1 probably interferes with the α-synuclein aggregation state by being incorporated as an integral part of the filament structure with the region around residue 94–96, maybe as a β-strand as hypothesized (or in alternative ways), and thereby, the previously exposed hydrophobic residues become surrounded by α-synuclein residues. PU and P2 could also form β-strands that form intramolecular interactions with the β-sheet filaments of aggregated α-synuclein but in a way that their exposed hydrophobic residues are not interacting with residues of surrounding β-strands of α-synuclein maybe by terminating the continuation of the filaments (as in the hypothesis). Alternatively, PU and P2 may have their hydrophobic residues remaining exposed but still interact with α-synuclein in another way, similar to what has been reported for α-helical peptides that bind oligomers and fibrils of α-synuclein [24], and cause disaggregation. However, the former hypothesis is more plausible because the very few and closely localized mutations in PU giving rise to P1 and P2 probably do not change much the interactions between the peptides and α-synuclein (since all three of them interact), but rather change their effects on the aggregation/disaggregation equilibrium.

Our in vitro results suggest that the C-terminal cytoplasmic domain of URG7 is implicated in interfering with α-synuclein aggregation. Furthermore, this study shows that URG7-derived peptides could be interesting therapeutic candidates for synucleinopathies, given that other peptides and polyphenols that modulate α-synuclein aggregation have shown promise for the treatment of neurodegenerative diseases [17,20,50,51,52]. For this purpose, future studies in cellular models are needed to characterize further disaggregation peptides derived from URG7, confirm that the full URG7 protein in a physiological environment is able to interact with and modulate α-synuclein structures, and determine its exact mechanism of action. Other factors involved in the UPR and associated with various diseases have been shown to act via different mechanisms [53], and the possible roles of URG7 in UPR could be several. However, considering that Parkinson’s is a prion-like disease, it is reasonable to think that the interaction between the C-terminal region of URG7 and α-synuclein shifts the conformational equilibrium of α-synuclein towards the soluble form, acting as a folder corrector. This new function suggested for URG7 may explain observations previously reported in which overexpressed URG7 causes a reduction in unfolded proteins, an attenuation of ER stress induced by tunicamycin in HepG2 cells, and a change in the UPR with an increase in survival pathways compared to pro-apoptotic pathways as well as in neuroblastoma cell line SH-SY5Y [2,54]. Taken together, the functions assigned to URG7 may suggest that it has a role in protein folding both in the ER lumen and the cytoplasmic compartment.

## 4. Materials and Methods

### 4.1. Design of Peptides

Peptides with a maximum length of 25 amino acids have been designed: AAIPGSLEPGNVRGRQGTGWNLVKS (called PU), AAIPGSLPGLNVRGRQGTGVTAVKS (identified as P1), and AAIPGSLPGLNVRGRQGVGWKLWKS (called P2). The peptide called P3 is the one identified by Meade et al. 2021 [17], and it was used as a positive control (KDGIVAGVKA). Peptides were commercially obtained from Pepscan Presto (Zuidersluisweg 2, 8243 RC Lelystad, The Netherlands).

### 4.2. α-Synuclein Aggregation

Expression and purification of α-synuclein were performed as previously reported [5]. After purification, dialysis, and freeze–drying, the protein is solubilized in 1.2 mL of phosphate buffer (PB) (KH_2_PO_4_ 3.3 mM e Na_2_HPO_4_ 3.8 mM, pH 6.8). α-synuclein concentration was determined spectrophotometrically at 280 nm using extinction coefficients 5960 M^−1^ cm^−1^ (https://web.expasy.org/protparam/) (accessed on 1 June 2022). α-synuclein solution at 0.7 mM was subjected to heating and stirring (200 rpm) at 45 °C for 2 h (HS45). Finally, the α-synuclein solution was aliquoted and frozen at −80 °C if not used immediately. Frozen aliquots of HS45 were thawed on ice for 30 min and then kept at room temperature for 30 min before use. Some experiments have also been conducted on α-synuclein used immediately after solubilization (S).

### 4.3. Western Blot Analysis

α-synuclein resuspended in Laemmli sample buffer (60 mM Tris–HCl pH 6.8, 10% glycerol, 2% SDS, 1% β-mercaptoethanol and 0.002% bromophenol blue) were loaded into sodium dodecyl sulfate–polyacrylamide gels (4–15%) for electrophoresis and electrophoretically transferred to nitrocellulose membranes (AmershamTM ProtranTM Nitrocellulose Blotting Membrane, 0.45 µm, GE Healthcare). The membranes were blocked in a saturation buffer (with 2% skim milk in TBS with 0.05% Tween 20, TBST) for 1 h at room temperature and then probed overnight at 4 °C with specific primary antibody 1:1000 anti- α-synuclein PA1-18264 (Invitrogen) in skim milk al 2% in TBS-T 0.05%. After washing, the membrane was incubated at room temperature for 1 h with appropriate horseradish peroxidase-conjugated secondary antibody. The signal was visualized by ECL™ Western Blotting Detection Reagents (GE Healthcare, Chicago, IL, USA) using a Chemidoc™ XRS detection system equipped with Image Lab 5.1 Software for image acquisition (BioRad 1000 Alfred Nobel Drive Hercules, California 94547 USA). 

### 4.4. Non-Denaturing Polyacrylamide Gel

A native polyacrylamide gel (15%) was used to evaluate the aggregation state of the α-synuclein. Samples (2 μg) were added with 10 μL of sample buffer (0.187 M Tris-HCl pH 6.8, 30% Glycerol, 80 μg/mL bromophenol blue). The running buffer consisted of Glycine (14.4 g/L) and Tris (3 g/L). Subsequently, the gel is stained with Coomassie Blue and decolorized in 10% acetic acid, 70% methanol, and 20% distilled H_2_O. Chemidoc™ XRS detection system equipped with Image Lab Software for image acquisition (BioRad) was employed.

### 4.5. Circular Dichroism

Circular dichroism (CD) spectra were recorded on a Jasco J-185 CD spectrophotometer (Jasco, 28600 Mary’s Court Easton, MD 21601) using a 0.1 cm cylindrical quartz cell. The spectra were recorded at 25 °C across wavelengths 190 nm to 250 nm, with a scanning speed of 100 nm/min, 1 nm bandwidth, a time-constant of 0.5 s, 20 mdeg of sensitivity, and a total number of 16 accumulations for each spectrum. Then, the baseline spectrum of the PB was subtracted, and spectra were smoothed using the Fourier transform. Peptides were used at 7 µM concentration; data were expressed in terms of the molar ellipticity per residue in units of deg × cm^2^ × dmol^−1^. and in θ(mdeg) for α-synuclein. Analysis of spectra for the evaluation of secondary structure content was performed with DichroWeb using the CONTINLL algorithm [26]. 

### 4.6. Differential Scanning Calorimetry

Calorimetric analyses were performed using a DSC Pyris calorimeter (Perkin Elmer, Waltham, MA, USA). The calorimeter was calibrated using cyclohexane and indium as standards, resulting in a temperature accuracy of 0.1 °C and an enthalpy accuracy of 0.2 J/g. High-resolution thermograms were recorded by steps of 0.01 °C. 

All the solutions (15 μL) were sealed in hermetic aluminum pans, and an empty pan was used as a reference. Solutions were cooling to −100 °C at 80 °C at 10 °C/min. 

The thermograms were recorded during two heating and cooling. The cooling and heating rates were chosen to obtain well-defined and reproducible results. Three replicates were performed for each sample. 

Information about structural and dynamic changes can be deduced from the amount of heat absorbed or emitted by the sample to the controlled temperature rate. Quantification of the different types of water molecules had been previously described [30].

### 4.7. FTIR Spectroscopy

FTIR-ATR spectra were performed using a Nicolet 5700 (Thermo Fisher Scientific, Waltham, MA, USA) equipped with an ATR accessory (Smart Orbit with a type IIA diamond crystal, refractive index 2.4) with a KBr beam splitter and an MCT/B detector. 

A volume of 2μL of solution was deposited on the ATR device and left to dry (for 15 min to 45 min, according to the different samples) to form a dry film before the acquisition of stable FTIR spectra. Measurements on the resulting dried films (64 accumulations) were recorded over the 4000–450 cm^−1^ region with a spectral resolution of 2 cm^−1^. The background spectrum was recorded before each experiment and subtracted from the sample spectrum. Three replicates were performed for each sample. 

Data were collected using Omnic 8.0. Spectra were baseline corrected smoothed (Savitzky–Golay’s method, 13 points). According to literature data [32] and in order to quantify the evolution of the secondary structures amount in the different α-synuclein samples, the decomposition of the Amide I was performed using the Peak Resolve function in the Omnic 8.0 Software. Based on the characteristic minima of the second derivative curves, the spectral range of 1600–1700 cm^−1^ was decomposed in eight bands, including one small marginal band (1605 cm^−1^) due to side-chain absorption whose positions are allowed to range between fixed limits (Table 3). According to literature data, it is noteworthy that β sheets structures absorb both at low (1615–1640 cm^−1^) and high (1675–1700 cm^−1^) wave numbers [55]; in contrast with the high wave numbers components corresponding to both intra- and inter- molecular β sheets, the low wave number components are useful to discriminate between these different structures. In the curve-fitting procedure, the combination of Gaussian-Lorentzian peak shapes was used for all the peaks, with a full-width-half-height (FWHH) ranging between 5 and 30 cm^−1^. 

A proportion of each component in the amide I band was computed as a fractional area of the corresponding peak divided by the sum of the areas of the peaks belonging to the amide I band.

Second-derivatives and Fourier-Self-Deconvolution (FSD) were also used to enhance the chemical information present in overlapping infrared absorption bands.

### 4.8. Emission Fluorescence Measurements

α-synuclein and the peptides were solubilized in PB and used at the concentrations of 70 µm and 105 µM, respectively. To evaluate the interaction of peptides PU and P2 with α-synuclein, spectra of peptides were recorded by setting the excitation wavelength at 275 nm and collecting the emission fluorescence in a range between 300 and 500 nm. Since both P1 and P3 do not contain aromatic amino acids to evaluate the interaction between P1 and P3 with α-synuclein, the spectra are recorded by setting the excitation wavelength at 264 nm at which α-synuclein fluoresces (due to phenylalanine in position 4 and 9 and tyrosine at positions 39, 125, 133 and 136) and fluorescence was collected between 270 and 420 nm. Spectra were recorded immediately (T0) and after 3 h (T3) using a Cary Eclipse Fluorescence Spectrophotometer (Agilent Technology, 5301 Stevens Creek Blvd Santa Clara, CA 95051 United States). As a control, it was verified that all the changes in fluorescence observed were not due to the addition of PB alone.

### 4.9. Thioflavin-T (ThT) Aggregation Assay

Thioflavin T (ThT) is able to selectively interact with the portions rich in β-sheets present in the amyloid aggregates and oligomers, resulting in an increase in the fluorescence intensity and a shift of the emission maximum. A concentration of 50 μM of ThT was added to 25 μM α-synuclein (HS45) without and with 25 μM of each peptide. Fluorescence was evaluated by fluorescence microscopy (Nikon Eclipse TS100, Nikon Europe B.V. Stroombaan 14, 1181 VX Amstelveen, The Netherlands) with the green light filter. As a control, no change in fluorescence was observed if PB was added. In addition, it was verified that the addition of thioflavin to freshly solubilized α-synuclein (S) did not emit any fluorescence. 

### 4.10. Transmission Electron Microscopy (TEM)

Carbon coating on 400 mesh copper grids (Agar Scientific Ltd, Unit 7, M11 Business Link, Parsonage Lane, Stansted, Essex CM24 8GF United Kingdom) was used. One drop of the α-synuclein at 0.7 mM concentration alone or spiked for 30 min with each peptide (105 μM) was applied to the grid and was left to stand for 4 min. Then grids were dabbing with Whatman paper, stained with 5 μL microfiltered uranyl acetate solution 2% (*w*/*v*) for 2 min, and finally dabbed with Whatman paper. The samples were analyzed using a FEI Tecnai G2 20 Twin Transmission Electron Microscope (FEI Italia S.r.l.-Via Monte Nero 84, 20135 Milano, Italy) at 120 kV. 

## Figures and Tables

**Table 1:** Thermal characteristics (temperature and enthalpy) of α-synuclein solutions (initial and after heating at 45 °C for 2 h); eutectic melting (Teut and ΔHeut); primary ice melting (Tm and ΔHm).

Solution	Teut °C	ΔHeut J/g	Tm °C	ΔHm J/g
Initial α-synuclein	−19.5	3.4	3.15	206.7
Heated α-synuclein	−21.1	2.5	5.4	272.2

**Table 2:** Thermal characteristics (temperature and enthalpy) of peptide solutions: clathrate-like structure formation (Tc and ΔHc), eutectic melting (Teut and ΔHeut), primary ice melting (Tm and ΔHm) from DSC analysis.

Peptide Solutions	Tc °C	ΔHc J/g	Teut °C	ΔHeut J/g	Tm °C	ΔHm J/g
P1	−57.3	−1.3	−23	3.2	3.3	271
P2	−72	−1.1	−22.6	3.9	3.4	268.4
P3	−69	−0.7	−22	3.9	2.3	261.2
PU	−74.4	−0.4	−21.3	5.1	2.7	269.4

**Table 3:** Parameters for the Amide I curve-fitting.

Shape	Peak Position (cm^−1^)	Accepted Limits	FWHH (cm^−1^)	Assignment
Gaussien/Lorentzien	1605	1595–1610	5–30	side chain
Gaussien/Lorentzien	1620	1615–1626	5–30	intermolecular β sheets
Gaussien/Lorentzien	1635	1630–1640	5–30	intramolecular β sheets
Gaussien/Lorentzien	1650	1645–1655	5–30	random coil/water
Gaussien/Lorentzien	1658	1655–1658	5–30	α helices/random
Gaussien/Lorentzien	1667	1665–1672	5–30	Turns, loops
Gaussien/Lorentzien	1680	1675–1685	5–30	β turns, β sheets
Gaussien/Lorentzien	1695	1690–1700	5–30	β sheets

## Data Availability

Data are contained within the article and Supplementary Materials.

## Supplementary Materials

The following supporting information can be downloaded at: https://www.mdpi.com/article/10.3390/ijms25021135/s1.

## References

[1] Ostuni A., Lara P., Armentano M.F., Miglionico R., Salvia A.M., Mönnich M., Carmosino M., Lasorsa F.M., Monné M., Nilsson I. (2013). The hepatitis B x antigen anti-apoptotic effector URG7 is localized to endoplasmic reticulum membrane. FEBS Lett..

[2] Armentano M.F., Caterino M., Miglionico R., Ostuni A., Pace M.C., Cozzolino F., Monti M., Millela L., Carmosino M., Pucci P. (2018). New insights on the functional role of URG7 in the cellular response to ER stress. Biol. Cell.

[3] Bisaccia F., Koshal P., Abruzzese V., Castiglione Morelli M.A., Ostuni A. (2021). Structural and Functional Characterization of the ABCC6 Transporter in Hepatic Cells: Role on PXE, Cancer Therapy and Drug Resistance. Int. J. Mol. Sci..

[4] Luo H., Li Q., Cao Y., Uitto J. (2020). Therapeutics Development for Pseudoxanthoma Elasticum and Related Ectopic Mineralization Disorders: Update. J. Clin. Med..

[5] Dandurand J., Ostuni A., Armentano M.F., Crudele M.A., Dolce V., Marra F., Samouillan V., Bisaccia F. (2020). Calorimetry and FTIR reveal the ability of URG7 protein to modify the aggregation state of both cell lysate and amylogenic α-synuclein. AIMS Biophys..

[6] Yoo G., Shin Y.K., Lee N.K. (2023). The Role of α-Synuclein in SNARE-mediated Synaptic Vesicle Fusion. J. Mol. Biol..

[7] McGlinchey R.P., Ni X., Shadish J.A., Jiang J., Lee J.C. (2021). The N terminus of α-synuclein dictates fibril formation. Proc. Natl. Acad. Sci. USA.

[8] Du H.N., Tang L., Luo X.Y., Li H.T., Hu J., Zhou J.W., Hu H.Y. (2003). A peptide motif consisting of glycine, alanine, and valine is required for the fibrillization and cytotoxicity of human alpha-synuclein. Biochemistry.

[9] Bengoa-Vergniory N., Roberts R.F., Wade-Martins R., Alegre-Abarrategui J. (2017). α-synuclein oligomers: A new hope. Acta Neuropathol..

[10] Guo H., Yi J., Wang F., Lei T., Du H. (2023). Potential application of heat shock proteins as therapeutic targets in Parkinson’s disease. Neurochem. Int..

[11] Serrano A., Qiao X., Matos J.O., Farley L., Cilenti L., Chen B., Tatulian S.A., Teter K. (2020). Reversal of Alpha-Synuclein Fibrillization by Protein Disulfide Isomerase. Front. Cell Dev. Biol..

[12] Oliveri V. (2019). Toward the discovery and development of effective modulators of α-synuclein amyloid aggregation. Eur. J. Med. Chem..

[13] Fields C.R., Bengoa-Vergniory N., Wade-Martins R. (2019). Targeting Alpha-Synuclein as a Therapy for Parkinson’s Disease. Front. Mol. Neurosci..

[14] Galkin M., Priss A., Kyriukha Y., Shvadchak V. (2023). Navigating α-Synuclein Aggregation Inhibition: Methods, Mechanisms, and Molecular Targets. Chem. Rec..

[15] Armiento V., Spanopoulou A., Kapurniotu A. (2020). Peptide-Based Molecular Strategies To Interfere with Protein Misfolding, Aggregation, and Cell Degeneration. Angew. Chem. Int. Ed. Engl..

[16] Martial B., Raîche-Marcoux G., Lefèvre T., Audet P., Voyer N., Auger M. (2020). Structure of a Parkinson’s Disease-Involved α-Synuclein Peptide Is Modulated by Membrane Composition and Physical State. J. Phys. Chem. B.

[17] Meade R.M., Watt K.J.C., Williams R.J., Mason J.M. (2021). A Downsized and Optimised Intracellular Library-Derived Peptide Prevents Alpha-Synuclein Primary Nucleation and Toxicity Without Impacting Upon Lipid Binding. J. Mol. Biol..

[18] Guo Q., Kawahata I., Jia W., Wang H., Cheng A., Yabuki Y., Shioda N., Fukunaga K. (2023). α-Synuclein decoy peptide protects mice against α-synuclein-induced memory loss. CNS Neurosci. Ther..

[19] El-Agnaf O.M., Paleologou K.E., Greer B., Abogrein A.M., King J.E., Salem S.A., Fullwood N.J., Benson F.E., Hewitt R., Ford K.J. (2004). A strategy for designing inhibitors of alpha-synuclein aggregation and toxicity as a novel treatment for Parkinson’s disease and related disorders. FASEB J..

[20] Popova B., Wang D., Rajavel A., Dhamotharan K., Lazaro D.F., Gerke J., Uhrig J.F., Hoppert M., Outeiro T.F., Braus G.H. (2021). Identification of Two Novel Peptides That Inhibit alpha-Synuclein Toxicity and Aggregation. Front. Mol. Neurosci..

[21] Agerschou E.D., Flagmeier P., Saridaki T., Galvagnion C., Komnig D., Heid L., Prasad V., Shaykhalishahi H., Willbold D., Dobson C.M. (2019). An engineered monomer binding-protein for α-synuclein efficiently inhibits the proliferation of amyloid fibrils. Elife.

[22] Ren Q., Jiang X., Zhang S., Gao X., Paudel Y.N., Zhang P., Wang R., Liu K., Jin M. (2022). Neuroprotective effect of YIAEDAER peptide against Parkinson’s disease like pathology in zebrafish. Biomed. Pharmacother..

[23] Rezaeian N., Shirvanizadeh N., Mohammadi S., Nikkhah M., Arab S.S. (2017). The inhibitory effects of biomimetically designed peptides on α-synuclein aggregation. Arch. Biochem. Biophys..

[24] Santos J., Gracia P., Navarro S., Peña-Díaz S., Pujols J., Cremades N., Pallarès I., Ventura S. (2021). α-Helical peptidic scaffolds to target α-synuclein toxic species with nanomolar affinity. Nat. Commun..

[25] Tuttle M.D., Comellas G., Nieuwkoop A.J., Covell D.J., Berthold D.A., Kloepper K.D., Courtney J.M., Kim J.K., Barclay A.M., Kendall A. (2016). Solid-state NMR structure of a pathogenic fibril of full-length human alpha-synuclein. Nat. Struct. Mol. Biol..

[26] Miles A.J., Ramalli S.G., Wallace B.A. (2022). DichroWeb, a website for calculating protein secondary structure from circular dichroism spectroscopic data. Protein Sci..

[27] Tompa K., Bánki P., Bokor M., Kamasa P., Lasanda G., Tompa P. (2009). Interfacial water at protein surfaces: Wide-line NMR and DSC characterization of hydration in ubiquitin solutions. Biophys. J..

[28] Dandurand J., Samouillan V., Lacabanne C., Pepe A., Bochicchio B. (2015). Water structure and elastin-like peptide aggregation. J. Therm. Anal. Calorim..

[29] Kamasa P., Bokor M., Pyda M., Tompa K. (2007). DSC approach for the investigation of mobile water fractions in aqueous solutions of NaCl and Tris buffer. Thermochim. Acta.

[30] Heys K.R., Friedrich M.G., Truscott R.J. (2008). Free and bound water in normal and cataractous human lenses. Investig. Ophthalmol. Vis. Sci..

[31] Cai Y., Lendel C., Osterlund L., Kasrayan A., Lannfelt L., Ingelsson M., Nikolajeff F., Karlsson M., Bergstrom J. (2015). Changes in secondary structure of alpha-synuclein during oligomerization induced by reactive aldehydes. Biochem. Biophys. Res. Commun..

[32] Ghosh D., Singh P.K., Sahay S., Jha N.N., Jacob R.S., Sen S., Kumar A., Riek R., Maji S.K. (2015). Structure based aggregation studies reveal the presence of helix-rich intermediate during alpha-Synuclein aggregation. Sci. Rep..

[33] Ghosh S., Kundu A., Chattopadhyay K. (2018). Small Molecules Attenuate the Interplay between Conformational Fluctuations, Early Oligomerization and Amyloidosis of Alpha Synuclein. Sci. Rep..

[34] Ghosh S., Sakshi Swain B.C., Chakraborty R., Tripathy U., Chattopadhyay K. (2020). A Novel Tool to Investigate the Early and Late Stages of alpha-Synuclein Aggregation. ACS Chem. Neurosci..

[35] Ladokhin A.S., Meyers R.A. (2000). Fluorescence Spectroscopy *in* Peptide *and* Protein Analysis. Encyclopedia of Analytical Chemistry.

[36] Uversky V.N., Eliezer D. (2009). Biophysics of Parkinson’s Disease Structure and Aggregation of alfa-synuclein. Curr. Protein Pept. Sci..

[37] Cremades N., Chen S.W., Dobson C.M. (2017). Structural Characteristics of alpha-Synuclein Oligomers. Int. Rev. Cell Mol. Biol..

[38] Buell A.K., Galvagnion C., Gaspar R., Sparr E., Vendruscolo M., Knowles T.P., Linse S., Dobson C.M. (2014). Solution conditions determine the relative importance of nucleation and growth processes in alpha-synuclein aggregation. Proc. Natl. Acad. Sci. USA.

[39] Tompa P., Bánki P., Bokor M., Kamasa P., Kovács D., Lasanda G., Tompa K. (2006). Protein-water and protein-buffer interactions in the aqueous solution of an intrinsically unstructured plant dehydrin: NMR intensity and DSC aspects. Biophys. J..

[40] Munishkina L.A., Henriques J., Uversky V.N., Fink A.L. (2004). Role of Protein−Water Interactions and Electrostatics in α-Synuclein Fibril Formation. Biochemistry.

[41] Aggarwal L., Biswas P. (2021). Hydration Thermodynamics of Familial Parkinson’s Disease-Linked Mutants of alpha-Synuclein. J. Chem. Inf. Model..

[42] Choi T.S., Han J.Y., Heo C.E., Lee S.W., Kim H.I. (2018). Electrostatic and hydrophobic interactions of lipid-associated alpha-synuclein: The role of a water-limited interfaces in amyloid fibrillation. Biochim. Biophys. Acta Biomembr..

[43] Qiao B., Jimenez-Angeles F., Nguyen T.D., Olvera de la Cruz M. (2019). Water follows polar and nonpolar protein surface domains. Proc. Natl. Acad. Sci. USA.

[44] Arya S., Singh A.K., Bhasne K., Dogra P., Datta A., Das P., Mukhopadhyay S. (2018). Femtosecond Hydration Map of Intrinsically Disordered alpha-Synuclein. Biophys. J..

[45] Chakraborty R., Dey S., Sil P., Paul S.S., Bhattacharyya D., Bhunia A., Sengupta J., Chattopadhyay K. (2021). Conformational distortion in a fibril-forming oligomer arrests alpha-Synuclein fibrillation and minimizes its toxic effects. Commun. Biol..

[46] Bhak G., Lee J., Kim T.H., Lee S., Lee D., Paik S.R. (2014). Molecular inscription of environmental information into protein suprastructures: Temperature effects on unit assembly of alpha-synuclein oligomers into polymorphic amyloid fibrils. Biochem. J..

[47] Cerf E., Sarroukh R., Tamamizu-Kato S., Breydo L., Derclaye S., Dufrene Y.F., Narayanaswami V., Goormaghtigh E., Ruysschaert J.M., Raussens V. (2009). Antiparallel beta-sheet: A signature structure of the oligomeric amyloid beta-peptide. Biochem. J..

[48] Dos Santos Rodrigues F.H., Delgado G.G., Santana da Costa T., Tasic L. (2023). Applications of fluorescence spectroscopy in protein conformational changes and intermolecular contacts. BBA Adv..

[49] Parui S., Jana B. (2019). Factors Promoting the Formation of Clathrate-Like Ordering of Water in Biomolecular Structure at Ambient Temperature and Pressure. J. Phys. Chem. B..

[50] Singh S.K., Dutta A., Modi G. (2017). α-Synuclein aggregation modulation an emerging approach for the treatment of Parkinson’s disease. Futur. Med. Chem..

[51] Kim Y.S., Lim D., Kim J.Y., Kang S.J., Kim Y.H., Im H. (2009). Beta-Sheet-breaking peptides inhibit the fibrillation of human alpha-synuclein. Biochem. Biophys. Res. Commun..

[52] Choi M.Y., Kim Y.S., Lim D., Kang S.J., Kim Y.H., Lee K., Im H. (2011). The hexapeptide PGVTAV suppresses neurotoxicity of human alpha-synuclein aggregates. Biochem. Biophys. Res. Commun..

[53] Lindholm D., Korhonen L., Eriksson O., Kõks S. (2017). Recent Insights into the Role of Unfolded Protein Response in ER Stress in Health and Disease. Front. Cell Dev. Biol..

[54] Nigro I., Miglionico R., Carmosino M., Gerbino A., Masato A., Sandre M., Bubacco L., Antonini A., Rinaldi R., Bisaccia F. (2024). Neuroprotective Effect of Antiapoptotic URG7 Protein on Human Neuroblastoma Cell Line SH-SY5Y. Int. J. Mol. Sci..

[55] Barth A. (2007). Infrared spectroscopy of proteins. Biochim. Biophys. Acta.

