# Supplementary material for: The 75–99 C-Terminal Peptide of URG7 Protein Promotes α-Synuclein Disaggregation
Source: Int J Mol Sci. 2024 Jan 17;25(2):1135. doi: 10.3390/ijms25021135 (PMC10816444; doi:10.3390/ijms25021135)
Supplement: Supplementary file 1 [file ijms-25-01135-s001.zip › Figure S1.pdf]

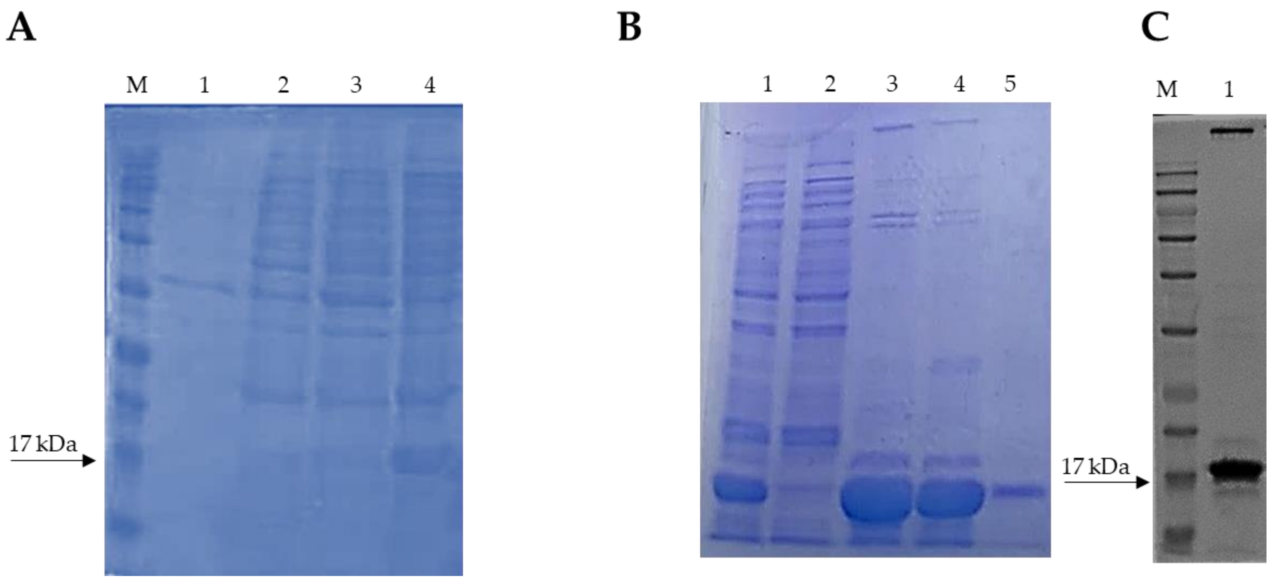

Supplementary Figure S1. Expression and purification of α–synuclein. (A) Protein expressed in BL21(DE3) cells as described in [5] and analyzed by SDS PAGE and Coomassie Blue staining. M: Marker Protein Ladder (5-245 kDa); lane 1: unsoluble proteins of non-induced bacteria; lane 2: soluble proteins of non-induced bacteria; lane 3: unsoluble proteins of IPTG induced bacteria; lane 4: soluble proteins of IPTG induced bacteria. (B) SDS-PAGE of 1: soluble fraction before purification from His TrapTM HP Column; 2: unbound proteins; 3-5: purified α–synuclein and (C) Western blotting analysis with polyclonal antibodies anti- α–synuclein PA1-1826. Images were analyzed with Chemidoc™ XRS detection system equipped with Image Lab Software for image acquisition (BioRad). The arrow indicates the molecular weight of the marker around which the monomeric form of recombinant α–synuclein is found.
