# Supplementary material for: The 75–99 C-Terminal Peptide of URG7 Protein Promotes α-Synuclein Disaggregation
Source: Int J Mol Sci. 2024 Jan 17;25(2):1135. doi: 10.3390/ijms25021135 (PMC10816444; doi:10.3390/ijms25021135)
Supplement: Supplementary file 1 [file ijms-25-01135-s001.zip › Table S1.pdf]

Supplementary Table S1. Thermal parameter of cold crystallization, (clathrate formation)

Sample	Tc °C	ΔHc J/g
P1 before and after heating	-57.3	-1.3
HS45+P1	----	------
P2 before and after heating	-72	-1.1
HS45+P2	-59	-1
P3 before and after heating	-69	-0.7
HS45+P3	-61	-1.5
PU before heating	-74.4	-0.4
PU after heating	-74	-0.7
HS45+PU	-62	-1
